# Structural and Pathogenic Impacts of *ABCA4* Variants in Retinal Degenerations—An In-Silico Study

**DOI:** 10.3390/ijms24087280

**Published:** 2023-04-14

**Authors:** Senem Cevik, Subhasis B. Biswas, Esther E. Biswas-Fiss

**Affiliations:** 1 Department of Medical and Molecular Sciences, College of Health Sciences, University of Delaware, 16 West Main Street, Suite 302 WHL, Newark, DE 19716, USA; ceviks@udel.edu (S.C.); biswassb@udel.edu (S.B.B.); 2 Ammon Pinizzotto Biopharmaceutical Innovation Center, 590 Avenue 1743, Newark, DE 19713, USA

**Keywords:** *ABCA4*, missense variant, VUS, protein modeling, pathogenicity prediction, AlphaFold2

## Abstract

The retina-specific ATP-binding cassette transporter protein ABCA4 is responsible for properly continuing the visual cycle by removing toxic retinoid byproducts of phototransduction. Functional impairment caused by ABCA4 sequence variations is the leading cause of autosomal recessive inherited retinal disorders, including Stargardt disease, retinitis pigmentosa, and cone-rod dystrophy. To date, more than 3000 *ABCA4* genetic variants have been identified, approximately 40 percent of which have not been able to be classified for pathogenicity assessments. This study examined 30 missense *ABCA4* variants using AlphaFold2 protein modeling and computational structure analysis for pathogenicity prediction. All variants classified as pathogenic (n = 10) were found to have deleterious structural consequences. Eight of the ten benign variants were structurally neutral, while the remaining two resulted in mild structural changes. This study’s results provided multiple lines of computational pathogenicity evidence for eight *ABCA4* variants of uncertain clinical significance. Overall, in silico analyses of ABCA4 can provide a valuable tool for understanding the molecular mechanisms of retinal degeneration and their pathogenic impact.

## 1. Introduction

The ATP-binding cassette, subfamily A, member 4 gene (OMIM #601691) encodes the retina-specific ABCA4 transmembrane protein. This 2273 amino acid-length protein is involved in recycling retinoid byproducts of phototransduction [1,2,3]. When impaired by a genetic variation, the ABCA4 protein fails to translocate these reactive substances, leading to toxic accumulation in the retina and eventually resulting in blinding diseases. *ABCA4* pathogenic variants cause several retinal autosomal recessive disorders, including Stargardt disease (STGD1, OMIM #248200) [1,2,3,4,5,6,7], cone-rod dystrophy (CORD3, OMIM #604116) [8,9,10], and retinitis pigmentosa (RP19, OMIM #601718) [8,10,11]. They are also linked to susceptibility to age-related macular degeneration [12,13,14,15,16]. With 3164 genetic variants reported in ClinVar (https://www.ncbi.nlm.nih.gov/clinvar/ (accessed on 31 March 2023)), *ABCA4* is the most polymorphic gene involved in retinal disease [17,18]. These variants include missense, nonsense, frameshift, splicing, and structural mutations, with missense mutations constituting 46%. At least half of the missense variations have been designated as variants of uncertain significance (VUS). In addition, 12% of the rest have conflicting interpretations based on the ClinVar database, one of the largest public clinical variation archives [19]. This unknown state of the pathogenicity of *ABCA4* genetic variants represents a serious barrier to future therapeutic options and prospective prognoses.

Historically, the vast majority of *ABCA4* variant classifications have had a basis in clinical phenotype. However, with the advent of NGS technologies, the number of genetic variants now exceeds those that can be classified based on clinical evidence. The pathology of the *ABCA4* genetic variations has been well associated with defects in the enzymatic function of the expressed protein [20,21,22,23,24,25,26,27,28], which makes functional studies the gold standard for pathogenicity prediction. However, experimental analysis of the immense amount of *ABCA4* sequence variations is impractical. On the other hand, structural bioinformatics can achieve substantial feasibility while inferring the effects of the variants on the protein structure and function. The recent guidelines of the American College of Medical Genetics and Genomics/Association for Molecular Pathology (ACMG/AMP) recommend adding computational evidence to the pathogenicity assessments of the variants in Mendelian diseases [29].

Many in silico tools have been developed for SNV pathogenicity prediction [30,31,32,33,34,35,36,37,38]. The first-generation bioinformatic tools evaluate variants primarily based on evolutionary conservation and the physicochemical properties of the substitutions [39]. Although these automated servers are highly beneficial in predicting the clinical significance of many sequence variants, their specificity remains challenging. Some of these tools have included structure-based features in their assessments to enhance the accuracy of the pathogenicity prediction [31,37,38,39,40]. However, the current gap is that the prediction tools usually do not provide users with detailed reports on the consequences of the variant on protein structure [39].

Here, we report a multifaceted computational protein structural analysis of 30 missense *ABCA4* variants found in the ClinVar clinical database. By applying computational approaches, including protein structure analysis using the ABCA4 cryo-EM structures [41,42,43] and the AlphaFold2-predicted protein models, we were able to obtain confirmation of pathogenicity or lack of pathogenicity for benign variants and gain insight into the significance of 10 missense *ABCA4* variants of uncertain significance. 

## 2. Results

### 2.1. AlphaFold2 Protein Modeling

To establish the confidence with which AlphaFold2 could model the ABCA4 protein structure and thus be suitable as a tool for exploring the consequences of genetic variants, we first modeled the ABCA4 WT protein. 

**Figure 1:**
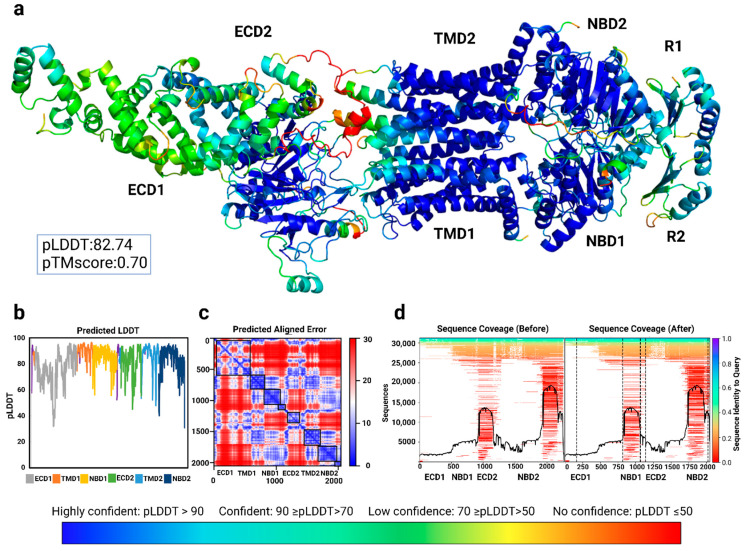
Confidence and quality of the full-length WT ABCA4 AlplaFold2 model generated in the ColabFold. (**a**) ABCA4 WT model colored based on the pLDDT (predicted Local Distance Difference Test) score in PyMol2. (**b**) Scatter plot illustrating the residual pLDDT. (**c**) Predicted aligned error (PAE), demonstrating the expected distance error in Å, which helps assess the confidence of the relative positions and the domain structures. ABCA4 domains are shown in black boxes: ECD1, TMD1, NBD1, R1 (regulatory subdomain of NBD1), ECD2, TMD2, NBD2, and R2 (regulatory subdomain of NBD2), from top-left to bottom-right, respectively. Except for the ECD1, all domains are well-defined in the AlphaFold2 model. (**d**) Sequence coverage before and after trimming the following residues: 164–208, 862–914, 1164–1203, 1279–1340, 2225–2231, and 2256–2273. The graphical data (**c**,**d**) have been auto-generated in the AlphaFold2advanced.ipynb notebook upon modeling [44]. The composite figure was created with BioRender.com.

The highly conserved nucleotide-binding domains of the ABCA4 (NBD1, NBD2) reached the best sequence coverage in the multiple sequence alignment (MSA) among the other domains (Figure 1d). Similarly, predicted aligned error (PAE) values are found to be higher in the nucleotide-binding (NBDs) and transmembrane domains (TMDs) than in the exocytoplasmic domains (ECDs) (Figure 1c). Overall, AlphaFold2 has well-defined the domains of the ABCA4 with high confidence.

We used the AlphaFold2_advanced.ipynb notebook by the MIT group [44] for the full-length protein models and trimmed parts that have not been able to be resolved by the cryo-EM studies (see Materials and Methods) [41,42,43]. These parts were covered in the domain-specific modeling (Supplementary Figure S1); however, they were random coils with lower confidence scores (Supplementary Figure S1 [45]). As these regions were absent from the experimental structures of the ABCA4 protein, trimming them did not affect the known domain organization while allowing a more confident, well-structured ABCA4 protein that was almost identical to the available experimental ABCA4 structures.

We obtained the WT and variant models with high confidence, as indicated by the pLDDT scores (pLDDT > 80) (Figure 1). The overall pLDDT score for the WT full-length protein was 82.74 (Figure 1a). Due to the lower sequence coverage and inherent structural flexibility [41,42,43], the exocytoplasmic domains of the ABCA4 protein showed the least confidence. The residual pLDDT scores based on the WT ABCA4 full-length model are presented in Table 1. All residues among the selected variants in the study, except for R1300Q and p.S2255I, gave pLDDT scores higher than 50, indicating the suitability of AlphaFold2 models for structure analysis.

### 2.2. In Silico Protein Structural Analysis and Pathogenicity Prediction of the ABCA4 Variants of Known Significance

This study aimed to create a workflow that could be used to predict a given *ABCA4* variant’s pathogenicity. To accomplish this, we created an in silico pipeline that combined protein structure analysis with standard informatics predictive tools, including allele frequency, evolutionary conservation, and pathogenicity prediction software (CADD, PolyPhen-2, REVEL, and MutPred-2). The in silico structure-based features include protein conformational changes and alignment scores (RMSD and TM-score), ∆∆G stability changes, clashing interactions, molecular bonds and other interactions, relative solvent accessibility (RSA), surface charges, and secondary structure elements. We first applied this method to the variants with known clinical significance to assess how in silico structural analysis of *ABCA4* variants correlates with pathogenicity. Twenty variants with classifications of “benign” or “pathogenic” in the public clinical database ClinVar were selected for the study.

#### 2.2.1. In Silico Analysis of *ABCA4* Benign Variants

**Figure 2:**
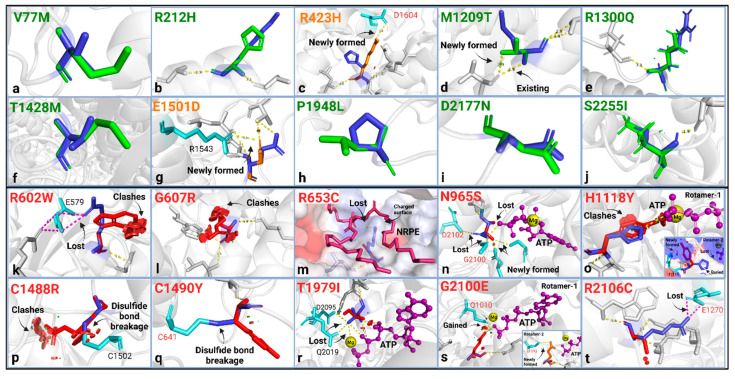
Highlights from the structural analysis of the benign (**a–j**) and pathogenic (**k–t**) *ABCA4* variants. WT residue (blue) and the substitution (green when there is no change, orange for mild change, and red for substantial change) were shown as sticks with their structural effects using the “mutagenesis” feature in PyMol2. No structural change was found in the benign variants, except for two mild structural changes: (**c**) R423H and (**g**) E1501D, which are predicted to introduce a new salt bridge to the structure. Pathogenic variants: (**k**) R602W; broken salt bridge with E579 and clashing. (**l**) G607R; clashing. (**m**) R653C; loss of polar contact with NRPE, altered surface properties around the substrate. (**n**) N965S; broken H-bonds with ATP, G2100, and D2102 (NBD1-NBD2 interaction in the ATP-bound state). (**o**) H1118Y; clashes with ATP or breaks polar content with ATP while introducing a new H-bond with H1119. (**p**,**q**) C1488R and C1490Y; disulfide bond breakage with C1502 and C641 (ECD1-ECD2 interaction), respectively. (**r**) T1979I; steric clashes with ATP, loss of polar contact with Mg, ATP, Q2019, and D2095. (**s**) G2100E; newly formed polar interactions with ATP, Mg, and Q1010 (rotamer-1) or with S1090 (rotamer-2), buried Gly replaced with a hydrophilic residue. (**t**) R2106C; broken salt bridge with E1270 in the NBD1 (domain-domain interaction). All other possible rotamers cause severe steric clashes. These analyses predicted the effects of side chains on the protein stereochemistry and bonding/interaction aspect of the variation and were mostly performed using the cryo-EM structures of the human ABCA4 [41,42,43,51,52,53,54,55,56,57]. Steric clashes were shown here for the highest probability rotamer but were only given when all possible rotamers resulted in clashing interactions. The bonds were given for the highest possible rotamer.

Using the in silico pipeline approach, we found that 8 of 10 benign *ABCA4* variants were structurally neutral, while 2 caused mild structural changes by introducing new H-bonds (Figure 2a-j), which may be stabilizing, as also predicted by the ∆∆G values (Table 1). The stability prediction suggested that 6 of the benign variants were neutral (−0.5 ≤ ∆∆G ≤ +0.5) (p.V77M, p.R1300Q, p.E1501D, p.P1948L, p.D2177N, and p.S2255I), 3 of them were stabilizing (∆∆G < −0.5) (p.R212H, p.H423R, and p.T1428M), and one variant, M1209T, was destabilizing (∆∆G > 0.05) (Table 1). The alignment scores, RMSD (Å) and TM-score (0–1), suggested a good alignment between the variants and the WT protein structure (Table 1). Standard informatics-based predictors, allele frequency, and evolutionary conservation agreed with the reported categorization of the variants as benign, except for the variant p.R212H, which was deemed pathogenic according to CADD, PolyPhen-2, and REVEL predictions. (Table S1). Despite this conflicting classification of the p.R212H in these predictive tools, all other qualifiers suggest that this variant is benign. We observed that protein structure analyses were consistent with standard informatics-based predictions for the known benign variants.

#### 2.2.2. In Silico Analysis of *ABCA4* Pathogenic Variants

Next, the in silico pipeline method was used to evaluate *ABCA4* variants previously classified as pathogenic in ClinVar. In our study, for the ten pathogenic variants, we found an array of structural alterations (Figure 2k–t). All the analyzed pathogenic variants were structurally damaging (Table 1). Three variants in the pathogenic group (p.R602W, p.G607R, and p.C1488R) caused steric clashing, four variants led to the loss of interactions with known ABCA4 substrates or ligands (p.R653C-NRPE-, p.N965S-ATP-, p.H1118Y-ATP-, and p.T1797I-ATP&Mg-), three variants (p.N965S, p.C1490Y, and p.R2106C) disrupted the interdomain interactions, and three (p.R602W, p.C1488Y, and p.T1979I) broke intrachain bonds (Figure 2k–t). The p.H1118Y variant was predicted to have two possible side chain rotamers, with one clashing with the ATP molecule and the other switching a buried residue to an exposed state (Figure 2o). Other variants that likely alter the surface properties are p.G607R, which replaces a buried Gly residue; p.C1488R, which exchanges an exposed residue with a buried residue; and G2100E, which replaces a buried Gly with a hydrophilic amino acid (Table S1). The ∆∆G stability calculation suggested that all pathogenic variants were destabilizing (∆∆G > 0.05), except for the H1118Y variant, which had a neutral stability (Table 1). While several measures contributed to the in silico evaluation of the variant protein structure, the RMSD and TM-score did not show a consistent conformational change or misalignment in most cases and thus did not appear valid as a standalone indicator of pathogenicity in the workflow. However, the variants p.R653C, p.N965S, and p.R2106C showed the highest RMSD (0.95, 0.98, and 0.95, respectively) and lowest TM-scores (0.82, 0.88, and 0.73, respectively) in this group, suggestive of conformational change (Table 1). We saw consistent agreement of the pathogenicity prediction with the structure-based and standard measures (Table S1).

### 2.3. In Silico Pipeline Analysis of ABCA4 Variants of Uncertain Significance

We next applied the in silico analysis to *ABCA4* variants classified by ClinVar as having uncertain significance (VUS). Seven of the ten *ABCA4* VUS (p.R20G, p.Y603F, p.L751P, p.T971P, p.G1558R, p.R2107H, and p.Y2165C) were predicted to be pathogenic by the informatics-based prediction tools, and all of these variants represent a substitution of highly conserved amino acids (Table 1 and Table S1) [58].

**Figure 3:**
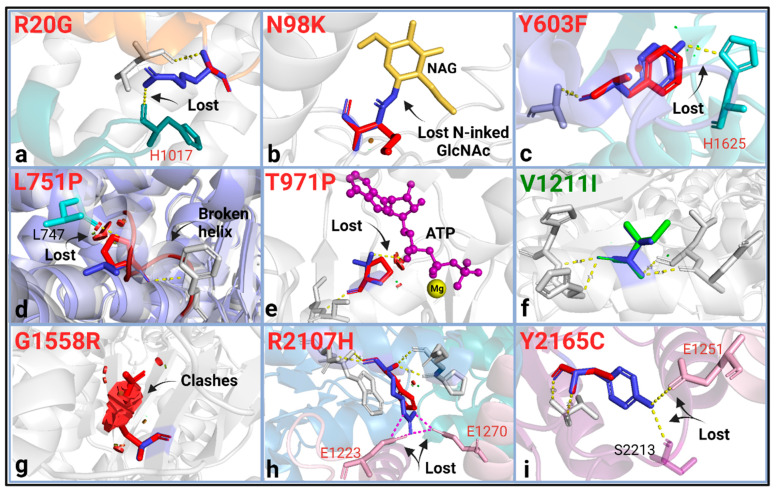
Protein structural analysis of the *ABCA4* VUS. WT residue (blue) and the substitution (green when there is no change, orange for mild change, and red for substantial change) were shown as sticks with their structural effects using the “mutagenesis” feature in PyMol2. (**a**) R20G; broken interchain H-bond (IH1 (orange)-NBD1 (green) interaction). (**b**) N98K; WT glycosylated Asn is replaced with Lys, which would otherwise be a neutral substitution. (**c**) Y603F; loss of an interchain H-bond (ECD1 (blue)-ECD2 (light green) interaction). (**d**) L751P; disrupted H-bond necessary for helix formation and steric clashes due to Pro not being able to accommodate into the TMD helix. (**e**) T971P; loss of polar contact with the ATP molecule (purple), steric clashes with ATP. (**f**) V1211I; no structural change was found. (**g**) G1558R; severe steric clashing. (**h**) R2107H; interdomain salt bridge breakage (NBD2 (blue)-NBD1-R1 (pink) interaction). (**i**) Y2165C; intra- and interchain H-bond breakage (NBD2-R2 (purple)-NBD1-R1 (pink) interaction). These analyses predicted the effects of side chains on the protein stereochemistry and bonding/interaction aspect of the variation and were mostly performed using the cryo-EM structures of the human ABCA4 [41,42,43,51,52,53,54,55,56,57]. Steric clashes were shown here for the highest probability rotamer but were only given when all possible rotamers resulted in clashing interactions. The bonds were given for the highest possible rotamer.

Five of these variants (p.R20G, p.Y603F, p.L751P, p.T971P, and p.G1558R) were absent in the population databases: GnomAD, ExAC, or 1000G, which provide a moderate degree of pathogenicity evidence (PM2) following ACMG/AMP guidelines [29]. The protein structure analysis found all seven predicted pathogenic variants to be structurally damaging (Table 1). The variants p.R20G, p.Y603F, p.R2107H, and p.Y2165C caused structural changes by breaking in domain-domain interactions (Figure 3a,c,h,i). The p.L751P variant introduced a Pro in the transmembrane helix, which is predicted to disrupt a helix by protein modeling. The P.T971P variant caused a loss of polar contact with the ATP molecule and introduced a buried Pro to the structure. The p.G1558R variant led to clashing interactions and replaced a buried Gly residue (Figure 3). All of these variants, except for p.Y603F, were thermodynamically destabilizing, as indicated by the predicted ∆∆G (Table 1).

The variants p.N98K and p.V1211I were deemed benign in CADD, PolyPhen-2, REVEL, and MutPred-2. The structural analysis agreed on the p.V1211I variant being neutral; however, it found p.N98K damaging by substituting an N-linked glycosylation site in the ECD1 (Figure 3).

The remaining *ABCA4* VUS in the study, c.5584G>A, was predicted to cause aberrant RNA splicing by altering the WT donor site according to the Human Splicing Finder tool (HSF-Pro) [47]. This variant, which changes G to A at the last nucleotide of exon 39 (the -1 of the canonical 5’ splice site), is predicted to introduce 3 new amino acids after Phe-1861 until it reaches premature termination. Therefore, although it was reported as c.5584G>A (p.Gly1862Ser), if the predicted effect is correct, a better denomination would be p.G1862fs*4. The protein modeling illustrates that this predicted truncated protein variant lost the last helix of the TMD2 and the entire NBD2 domain (Supplementary Figure S2). 

## 3. Discussion

Evidence from computational protein structure analysis can aid in predicting the clinical significance of the sequence variants in genetic diseases. Our goal was to create a compact yet effective, systematic, in silico structure-based approach to predict the pathogenicity of the *ABCA4* VUS. To assess their reliability, we first applied this method to variants with known pathogenicity. Our results indicated that our in silico predictions align well with the reported pathogenicity of the *ABCA4* variants in the clinical databases and the previous functional studies of these variants. Using this approach, we obtained pathogenicity evidence for variants previously reported as having undetermined significance.

Functional studies of the ABCA4 protein are important to understand how genetic variations affect the protein’s activity and how this, in turn, impacts retinal function. Functional studies can help confirm a genetic variant’s pathogenicity, which is essential for making an accurate diagnosis and providing appropriate treatment. Five of the *ABCA4* variants in our pathogenic group (p.R602W, p.R653C, p.N965S, p.C1488R, and p.C1490Y) and two variants from the benign group (p.D2177N) have been previously studied functionally [20,21,25,26,27,28,43]. All variants in the pathogenic group have been reported with functional defects [20,25,26,27,43]. The p.D2177N benign variant was shown to have a higher basal and retinal-stimulated ATPase activity than the wild-type ABCA4 protein [21]. Thus, our results agree well with previous functional studies (Table 1 and Table S1). It is worthwhile to note that conducting functional studies on a large number of genetic variants can be a challenging task. Traditional biochemical methods, such as enzyme ATPase assays and protein expression and purification, can be time-consuming and require significant resources. One way to address this challenge is to use computational methods to forecast the functional impact of genetic variants. Computational tools can suggest whether a variant is likely deleterious based on the impact of the amino acid change on protein structure and function and can help prioritize variants for functional studies.

Assessing the pathogenicity of *ABCA4* VUS is an active area of research, and several methods have been developed to predict the impact of VUS on protein function and disease risk. It is important to note that no single method can definitively classify VUS as pathogenic or benign. Rather, a combination of methods and expert interpretation is needed to make a conclusive assessment. The American College of Medical Genetics and Genomics (ACMG) and the Association for Molecular Pathology (AMP) have developed guidelines for the interpretation of genetic variants, including criteria for assessing VUS’s pathogenicity [29]. Our pipeline approach combines computational prediction tools, in silico modeling, and clinical correlation to assess pathogenicity. Our prediction defined eight of the *ABCA4* VUS as pathogenic and one VUS (p.V1211I) as benign (Table 1 and Table S1), which provided supporting evidence (PP3 and BP4, respectively) for these variants following the ACMG/AMP guidelines [29]. One variant (p.N98K), on the other hand, was found structurally deleterious yet predicted to be benign in all informatics-based tools (Figure 3b, Table S1). One variant in the VUS group, p.R2107H, has been shown to adversely affect the basal ATPase activity in functional in vitro experiments [26]. Our computational structure analysis demonstrating the molecular effect of this variant on the NBD1-NBD2 domain-domain interaction overlaps with this previous finding of the negative impact on the basal but not retinal-stimulated ATPase activity of the ABCA4 protein [26].

Some regions in the ABCA4 protein could not be modeled at full length using AlphaFold2. These regions were not present in any of the available cryo-EM structures of the human ABCA4 protein. Domain-specific modeling of these locations gave random coils with lower confidence scores. The absence of any defined secondary structure in these regions might be a limitation of the modeling approach, or they may be authentic random coils. In the ClinVar database, there are currently 93 missense ABCA4 variants falling within these regions. A limited number of these variants have been computationally analyzed using classical informatics-based tools [59,60,61] or are reported as part of the submission to the databases, such as ClinVar. Nearly 90% of these variants are VUS (https://www.ncbi.nlm.nih.gov/clinvar/ (accessed on 31 March 2023)).

ABC transporter proteins are known to transition between various distinct conformations, and their ability to do so is critical for their function. [41,42,43,62,63]. While the 3D structures and the AlphaFold2 prediction models can only capture one of these conformations, it is possible that the effect of a genetic variant could be linked to a property present in a different conformation or affect the protein’s overall dynamics. In this study, we did not evaluate the dynamic behavior of the ABCA4 protein. However, it is important to consider the impact of a variant in the context of these multiple conformations to fully understand its functional consequences, particularly for variants that are found to be free from other types of structural damage.

Several methods have been developed and used to compare two protein structures to evaluate the accuracy of predicted models and predict the structural effects of mutations, all with advantages and limitations [64,65,66,67]. For instance, the root-mean-square deviation (RMSD) is one of the most commonly used methods for computing backbone alignment scores, but this distance-based method is size-dependent and has low confidence in certain circumstances [48,68]. Therefore, when assessing the impacts of variants on protein structure, it might be best to use multiple combined methods. The alignment scores in this study, RMSD and TM-score, did not strictly correlate with the known pathogenicity of the *ABCA4* variants. Although these measures indicated no deleterious effect for the benign variants (low RMSD, high TM-score), most of the pathogenic variants also gave good alignment scores (Table 1). The RMSDs ranged from 0.302 to 1.915 Å, and the TM-scores were between 0.7278 and 0.9865. We did not set a threshold for these scores for pathogenicity, yet the variants with higher RMSD and lower TM-scores are R653C, N965S, and R2106C in the pathogenic category, and L751P and D915N in the VUS category. 

To our surprise, one VUS in this study, c.5584G>A, was classified as a missense variant in the public databases (p.Gly1862Ser), and yet the online splicing prediction tool HSF found that it likely affects splicing [47]. This result suggests that the prediction of the effects of genetic variation on an mRNA level should be included when assessing variant pathogenicity.

In summary, our findings suggest that in silico structural analysis, particularly when combined with other bioinformatics-based tools, can provide insight into *ABCA4* variants’ impact on the molecular mechanism, highlight potential variants of interest for functional characterization, and aid in pathogenicity prediction. Our findings demonstrate that computational approaches can forecast the structural aberrations arising from *ABCA4* genetic variants. Overall, the findings of this study suggest that an in silico pipeline approach can be widely used to prioritize variants of uncertain significance. 

## 4. Materials and Methods

### 4.1. Curation of the ABCA4 Variants from Databases and Pathogenicity Prediction 

The missense *ABCA4* variants were retrieved from the ClinVar database. There are currently only ten benign missense *ABCA4* variants in this clinical database. All ten of them were included in this study. Ten pathogenic and ten VUS *ABCA4* missense variants were selected to be distributed among the ABCA4 protein domains. We have included conventional pathogenicity prediction tools, evolutionary conservation information, and another well-accepted classifier in the variant pathogenicity assessment, allele frequency, to obtain “multiple lines of computational evidence” [29]. We have selected CADD [37], PolyPhen-2 [31], REVEL [36], and MutPred-2 [38] for the pathogenicity prediction of the variants due to their reported superior performances in the literature [39,69,70,71]. The Combined Annotation-Dependent Depletion (CADD) tool outputs “PHRED-scaled” scores, ranging from 0 to 99, with higher scores indicating a higher likelihood of pathogenicity. The possible outputs of Polyphen-2 are benign, possibly damaging, and probably damaging, along with a probability score. The REVEL platform gives pre-computed scores ranging from 0 (benign) to 1, with a pathogenicity threshold of 0.5. Similarly, the MutPred-2 scale is from 0 to 1, with increasing pathogenicity. Despite their known clinical significance, even benign and pathogenic variants were subjected to in silico pathogenicity prediction to validate the accuracy of these tools. The ConSurf web server was used to analyze the evolutionary conservation of the variant locations (Table S1) [58]. Allele frequencies of the variants were obtained from the GnomAD database [46]. All missense variants were analyzed for possible splicing defects in the Human Splicing Finder Ver. 3.1 (HSF-Pro from Genomnis) [47].

### 4.2. AlphaFold2 Protein Modeling

The WT and variant ABCA4 proteins were modeled with AlphaFold2 deep-learning-based protein modeling software in Google Colab. We used the AlphaFold.ipynb notebook by Deepmind [72] for domain-specific models and the AlphaFold2_advanced.ipynb notebook by the MIT group [44] for the full-length protein models as it allows for a trimming option; otherwise, modeling 2273 amino acid-length ABCA4 was not feasible. We trimmed the following residues: 164–208, 862–914, 1164–1203, 1279–1340, 2225–2231, and 2256–2273, which were low-confidence random coils in the domain-specific protein models. All available cryo-EM structures lack these regions [41,42,43]. Aside from that, we followed the set parameters on the ColabFold notebooks and used the highest confidence model (rank 1) among the generated models for the WT and each variant model for the downstream analyses.

The predicted structures were refined for energy minimization using the “repair object” command in the FoldX plugin for YASARA [49,50].

### 4.3. Protein Structure Analysis

The variants were analyzed in terms of backbone alignment, steric clashing interactions, bonding and nonbonding interactions, and surface properties (surface charge and relative solvent accessibility (RSA)) in the PyMOL2 software (The PyMOL Molecular Graphics System, Version 2.0 Schrödinger, LLC, New York, NY, USA). The ABCA4 variant protein models were superposed onto the WT model to detect conformational and secondary structure alterations and to obtain alignment scores. RMSDs were computed in PyMol2, and TM-scores were obtained from a web server by Zhang Lab [48]. 

The clashing interactions were tested not only on the refined computational models but also on all the available experimental structures of human ABCA4 to ascertain that they are not artifacts of predicted protein modeling. For the intramolecular and small molecule interactions (ATP, Mg+2, and the NRPE substrate), ATP-bound and substrate-bound cryo-EM structures were utilized (PDB ID: 7lkz, 7e7q, 7e7o, and 7m1q [51,52,53,54,55,56,57]). The APBS (Adaptive Poisson-Boltzmann Solver) plugin [73] was used to analyze the possible alterations of electrostatic properties and surface charge in Pymol2.

Potential destabilizing effects of the variants were predicted using the Gibbs free energy change ∆∆G method using the FoldX plugin in the YASARA program [49,50]. The refined full-length ABCA4 AlphaFold2 structure was mutated in the same program for the ∆∆G calculations.

We grouped variants into three categories, deleterious, mild, or neutral, according to their structural impact on the protein models.

## 5. Conclusions

Understanding the consequences of genetic variants is the crucial first step in the clinical management of inherited diseases. This report presents the pathogenicity prediction of ten missense *ABCA4* VUS using a multifaceted in silico protein structure analysis approach. The study findings suggest that protein modeling and computational structural analysis can aid in elucidating the structural and functional consequences of genetic *ABCA4* variations at the protein level.

## Figures and Tables

**Table 1:** *ABCA4* missense variants with their corresponding pathogenicity and in silico structural analysis results.

Clinical Significance	*ABCA4* Variant	Previous Functional Studies	Allele Freq.	Predicted Pathogenicity (Collective)	RMSD (Å)	TM-Score	In Silico ∆∆G (kcal/mol)	Predicted Effect on Structure
**Benign**	c.229G > A(p.V77M)	-	-	Benign	0.534	0.9333	−0.09	Neu
	c.635G > A(p.R212H)	-(R212C: ↓ ATPase [26])	4.24 × 10^−2^	Pathogenic ^a^	0.793	0.9372	−5.19	Neu
	c.1268A > G(p.H423R)	-	2.56 × 10^−1^	Benign	0.647	0.9333	−2.3	Mild
	c.3626T > C(p.M1209T)	-	3.03 × 10^−3^	Benign	0.539	0.9034	+0.71	Neu
	c.3899G > A(p.R1300Q)	-	6.70 × 10^−3^	Benign	0.775	0.9507	+0.007	Neu
	c.4283C > T(p.T1428M)	-	4.44 × 10^−3^	Benign	0.660	0.9865	−0.85	Neu
	c.4503G > C(p.E1501D)	-	1.12 × 10^−3^	Benign	0.326	0.9199	−0.38	Mild
	c.5843_5844inv (p.P1948L)	-	3.14 × 10^−2^	Benign	0.405	0.9652	+0.48	Neu
	c.6529G > A (p.D2177N)	↑ ATPase [21]	1.09 × 10^−2^	Benign	0.554	0.9683	−0.02	Neu
	c.6764G > T (p.S2255I)	-	1.59 × 10^−1^	Benign	0.302	0.9619	+0.13	Neu
Pathogenic/ Likely pathogenic **(P/LP)**	c.1804C > T:p(R602W)	↓ ATPase^26^ Mislocalization [26,27]	4.38 × 10^−5^	Pathogenic	0.507	0.9034	+16.56	Del
	c.1819G > C (p.G607R)	-	2.83 × 10^−5^	Pathogenic	0.588	0.9447	+67.4	Del
	c.1957C > T (p.R653C)	↓ Retinal-stim. ATPase [25,43]	1.61 × 10^−5^	Pathogenic	**0.953**	**0.8201**	+0.8	Del
	c.2894A > G:p(N965S)	↓ Expression, ↓ ATPase [20,26]	1.35 × 10^−4^	Pathogenic	**0.979**	**0.8819**	+1.1	Del
	c.3352C > T (p.H1118Y)	-	1.0 × 10^−5^	Pathogenic	0.487	0.9513	+0.15	Del
	c.4462T > C (p.C1488R)	↓ ATPase^20^, ↓ ATR binding [28]	8.20 × 10^−6^	Pathogenic	0.529	0.9655	+21.16	Del
	c.4469G > A (p.C1490Y)	Mislocalization, ↓ ATPase [26]	5.91 × 10^−5^	Pathogenic	0.345	0.9530	+41.76	Del
	c.5936C > T (p.T1979I)	-	-	Pathogenic	0.630	0.9698	+2.74	Del
	c.6299G > A (p.G2100E)	-	-	Pathogenic	0.450	0.9139	+2.85	Del
	c.6316C > T (p.R2106C)	-	1.31 × 10^−4^	Pathogenic	**0.945**	**0.7278**	+3.24	Del
**VUS**	c.58A > G (p.R20G)	-	-	Pathogenic	0.410	0.9429	+2.23	Del
	c.294C > G (p.N98K)	-	1.10 × 10^−4^	Benign	0.503	0.9547	+0.49	Del
	c.1808A > T (p.Y603F)	-	-	Pathogenic ^b^	0.520	0.9567	−0.75	Del
	c.2252T > C (p.L751P)	-	-	Pathogenic ^c^	**1.019**	**0.7705**	+9.49	Del
	c.2911A > C (p.T971P)	-	-	Pathogenic	0.559	0.9477	+0.68	Del
	c.3631G > A (p.V1211I)	-	-	Benign	0.379	0.9606	−3.27	Neu
	c.4672G > A (p.G1558R)	-	-	Pathogenic	0.625	0.9339	+74.05	Del
	c.5584G > A (p.G1862S)	-	-	Pathogenic ▪	0.628	0.9181	N/A	**pLoF**
	c.6320G > A:p(R2107H)	↓ ATPase^26^	2.03 × 10^−3^	Pathogenic	0.789	0.9615	+1.56	Del
	c.6494A > G (p.Y2165C)	-	6.57 × 10^−6^	Pathogenic	0.677	0.9491	+1.38	Del

Reference genome assembly: GRCh38:Chr1:83457325-104273917; reference transcript: NM_000350.3. Allele frequencies are based on the Genome Aggregation Database (gnomAD) v2.1.1 [46]. Functional studies: ↓ Reduced ATPase activity, ↑ Increased ATPase activity. Pathogenicity prediction: a variant is pathogenic when PolyPhen-2 predicts probably damaging or possibly damaging, REVEL and MutPred-2 score 0.5, and CADD ≥ 20. ^a^: The variant R212H was predicted to be pathogenic in the CADD, PolyPhen-2, and REVEL but neutral in the MutPred-2. ^b^,^c^: The variants Y603F and L751P were predicted to be damaging in the CADD, MutPred-2, and REVEL but benign in the PolyPhen-2. For these three conflicting outputs, three agreeing results were given here. ▪ The conventional pathogenicity prediction of c.5584G>A (p.Gly1862Ser) was conducted based on the reported nomenclature G1862S. However, this variant was predicted to cause aberrant splicing by broaching the WT donor splice site (HSF-Pro), likely leading to a premature termination: p.G1862fs*4. Therefore, the predicted pathogenicity may not reflect the true variant impact [47]. Residual pLDDT ≤ 50 for Arg-1300 (based on the domain-specific model) and Ser-2255, suggesting a lack of confidence as indicated by **ǂ**, should be interpreted with caution. RMSD (Å) (the root-mean-square deviation) and TM-score are based on the structural comparison of the predicted variant models with the predicted WT ABCA4 model. 0.0 < TM-score < 1, where 0 is 100% alignment [48]. We did not set a threshold for pathogenicity but showed the less well-aligned structures in this dataset in bold. Destabilizing variants were predicted using ∆∆G Gibbs free energy change calculations in the FoldX plugin for YASARA [49,50]. In the last column, the predicted effect on the structure is solely based on the protein structures and predicted models and is independent from the rest of Table 1, e.g., alignment scores and ∆∆G. ECD: exocytoplasmic domain; NBD: nucleotide-binding domain; TMD: transmembrane domain; IH: intracellular α-helix; VUS: variants of uncertain significance; Neu: neutral; Del: deleterious; pLoF: The predicted effect is a complete loss of function. For the detailed pathogenicity prediction and the protein structure analysis results, see Table S1.

## Data Availability

Additional data is available on request from the corresponding author.

## Supplementary Materials

The supporting information can be downloaded at: https://www.mdpi.com/article/10.3390/ijms24087280/s1.
