# Supplementary material for: Structural and Pathogenic Impacts of ABCA4 Variants in Retinal Degenerations—An In-Silico Study
Source: Int J Mol Sci. 2023 Apr 14;24(8):7280. doi: 10.3390/ijms24087280 (PMC10138569; doi:10.3390/ijms24087280)
Supplement: Supplementary file 1 [file ijms-24-07280-s001.zip › ijms-2272530-supplementary.pdf]

Table S1. Detailed analysis and pathogenicity prediction of the *ABCA4* variants.

	ABCA4 Variant	Domain	Allele Freq.	ConSurf Score	Functional Study	CADD Score	Prediction PolyPhen-2	Prediction REVEL	Prediction MutPred-2	AlphaFold2 Residual pLDDT Score	RMSD (Å)	TM-Score	*In silico* ΔΔG (kcal/mol)	Steric Clashes	RSA (WT vs. Mt)	Structural Change	Predicted effect on structure
1	c.229G>A(p.Val77Met)	ECD1	-	5	-	10.80	B	0.194	0.194	69.95	0.534	0.9333	-0.09	No	62% - 69%	-	Neu
2	c.635G>A(p.Arg212His)	ECD1	4.24×10^{-2}	1	**(R212C: lower ATPase [26])**	23.9	**PrD**	**0.716**	0.309	63.04	0.793	0.9372	-5.19	No	53% - 71%	-	Neu
3	c.1268A>G(p.His423Arg)	ECD1	2.56×10^{-1}	5	-	5.953	B	0.293	0.095	83.28	0.647	0.9333	-2.3	No	17% - 20%	Newly formed salt bridge with D1604	Mild
4	c.3626T>C(p.Met1209Thr)	NBD1-R1	3.03×10^{-3}	5	-	8.439	B	0.16	0.092	87.49	0.539	0.9034	+0.71	No	40% - 33%	-	Neu
5	c.3899G>A(p.Arg1300Gln)*	NBD1	6.70×10^{-3}	2	-	5.193	B	0.215	0.052	24.81ǂ	0.775	0.9507	+0.007	No!	-	-	Neu
6	c.4283C>T(p.Thr1428Met)	ECD2	4.44×10^{-3}	1	-	3.688	B	0.25	0.072	87.07	0.660	0.9865	-0.85	No	49% - 51%	-	Neu
7	c.4503G>C(p.Glu1501Asp)	ECD2	1.12×10^{-3}	3	-	9.501	B	0.209	0.201	85.49	0.326	0.9199	-0.38	No	34% - 39%	Newly formed salt bridge with R1543	Mild
8	c.5843_5844inv(p.Pro1948Leu)	NBD2	3.14×10^{-2}	1	-	-	B	N/A	0.274	74.38	0.405	0.9652	+0.48	No	92% - 96%	-	Neu
9	c.6529G>A (p.Asp2177Asn)	NBD2-R2	1.09×10^{-2}	7	Higher ATPase than WT [21]	14.33	B	0.313	0.112	55.64	0.554	0.9683	-0.02	No	94% - 74%	-	Neu
10	c.6764G>T (p.Ser2255Ile)	NBD2	1.59×10^{-1}	3	-	5.628	B	0.2	0.050	42.5ǂ	0.302	0.9619	+0.13	No	-	-	Neu
11	c.1804C>T :p(Arg602Trp)	ECD1	4.38×10^{-5}	8	Lower ATPase [26] Mislocalization [26,27]	24.7	PoD	0.934	0.564	87.6	0.507	0.9034	+16.56	Yes	16% - 18%	Breakage of a salt bridge with E579	Del
12	c.1819G>C (p.Gly607Arg)	ECD1	2.83×10^{-5}	9	-	25.9	PrD	0.952	0.890	90.03	0.588	0.9447	+67.4	Yes	0% - 3%	Steric clashes, Changes in the secondary structure, buried Gly replaced	Del
13	c.1957C>T (p.Arg653Cys)	TMD1	1.61×10^{-5}	6	Lower retinal-stimulated ATPase [25,43]	28.7	PrD	0.852	0.788	75.59	**0.953**	**0.8201**	+0.8	No	51% - 49%	Loss of polar contact with the NRPE	Del
14	c.2894A>G:p(Asn965Ser)	NBD1	1.35×10^{-4}	9	Lower expression, lower ATPase [20,26]	24.8	PrD	0.779	0.841	82.07	**0.979**	**0.8819**	+1.1	Mild	9% - 15%	Breakage of H-bonds with ATP, G2100, D2102 (NBD1-2 interaction in the ATP-bound state	Del
15	c.3352C>T (p.His1118Tyr)	NBD1	1.0×10^{-5}	9	-	27.2	PrD	0.979	0.878	83.06	0.487	0.9513	+0.15	Yes	**8% - 30%**	Clashes with ATP or breaks polar content with ATP while introducing new H-bond with H1119, buried switched to exposed	Del
16	c.4462T>C (p.Cys1488Arg)	ECD2	8.20×10^{-6}	9	Lower retinal-stimulated ATPase [20], Lower ATR binding [28]	25.5	PoD	0.93	0.889	88.6	0.529	0.9655	+21.16	Yes	**33% - 12%**	Disulfide Bond breakage with C1502, exposed switched to buried	Del
17	c.4469G>A (p.Cys1490Tyr)	ECD2	5.91×10^{-5}	9	Mislocalization, lower ATPase [26]	28.7	PrD	0.95	0.857	88.89	0.345	0.9530	+41.76	Mild	76% - 72%	Disulfide Bond breakage with C641 (ECD1-2 interaction)	Del
18	c.5936C>T (p.Thr1979Ile)	NBD2	-	9	-	28.9	PrD	0.961	0.901	89.13	0.630	0.9698	+2.74	Mild	57% - 51%	Steric clashes with ATP, loss of polar contact with Mg, ATP, Q2019, D2095	Del
19	c.6299G>A (p.Gly2100Glu)	NBD2	-	9	-	27.8	PrD	0.952	0.841	74.3	0.450	0.9139	+2.85	Mild	19% - 14%	Newly formed polar interaction with ATP, Mg, and Q1010 or with S1090, buried Gly replaced with a hydrophilic residue	Del
20	c.6316C>T (p.Arg2106Cys)	NBD2	1.31×10^{-4}	9	-	31	PrD	0.97	0.908	85.93	**0.945**	**0.7278**	+3.24	Mild	5% - 12%	Loss of salt bridge with E1270 in the ATP-bound state (ECD1-2 interaction)	Del
21	c.58A>G (p.Arg20Gly)	IH1	-	9	-	28.1	PrD	0.915	0.905	89.54	0.410	0.9429	+2.23	No	43% - 68%	Breakage of an H-bond with H1017 (Interchain interaction)	Del
22	c.294C>G (p.Asn98Lys)	ECD1	1.10×10^{-4}	7	-	16.93	B	0.487	0.326	69.03	0.503	0.9547	+0.49	No	65% - 55%	Loss of an N-linked Glycosylation site	Del
23	c.1808A>T (p.Tyr603Phe)	ECD1	-	9	-	26.1	B	**0.961**	**0.806**	91.27	0.520	0.9567	-0.75	No	4% - 5%	Breakage of an H-bond with H1625 (ECD1-2 interaction)	Del
24	c.2252T>C (p.Leu751Pro)	TMD1	-	7	-	24.8	B	**0.817**	**0.891**	94.83	**1.019**	**0.7705**	+9.49	Yes	3% - 9%	Helix breakage in the TMD, buried Pro introduced	Del
25	c.2911A>C (p.Thr971Pro)	NBD1	-	9	-	28.3	PrD	0.997	0.886	90.24	0.559	0.9477	+0.68	Yes	19% - 22%	Loss of polar contact with ATP, buried pro introduced	Del
26	c.3631G>A (p.Val1211Ile)	NBD1-R1	-	4	-	8.244	B	0.156	0.092	87.7	0.379	0.9606	-3.27	No	30% - 31%	-	Neu
27	c.4672G>A (p.Gly1558Arg)	ECD2	-	9	-	27.3	PrD	0.921	0.902	93.1	0.625	0.9339	+74.05	Yes	0% - 0%	Severe clashes, buried Gly replaced	Del
28	c.5584G>A (p.Gly1862Ser)	TMD2	-	N/A	-	34	PrD▪	0.661▪	0.679▪	59.96▪	0.628	0.9181	N/A	No	N/A	Predicted to affect splicing: Broken WT Donor (HSF Pro). Premature termination: p.G1862fs*4	pLoF
29	c.6320G>A:p(Arg2107His)	NBD2	2.03×10^{-3}	9	Lower ATPase [26]	29.3	PrD	0.88	0.759	83.56	0.789	0.9615	+1.56	Mild	8% - 7%	Breakage of salt bridges with E1223 and E1270 (NBD1-2 interaction)	Del
30	c.6494A>G (p.Tyr2165Cys)	NBD2-R2	6.57×10^{-6}	9	-	26.7	PrD	0.943	0.852	82.94	0.677	0.9491	+1.38	No	5% - 12%	Breakage of H-bonds with S2213 and E1251 in the ATP-bound state (R1-2 interaction)	Del

Reference genome assembly: GRCh38:Chr1:83457325-104273917, Reference Transcript: NM_000350.3. ConSurf Score: Amino acid evolutionary conservation scoring is based on the ConSurf web server analysis, and the range is between 1-9 with increasing conservation [47]. Allele frequencies are based on The Genome Aggregation Database (gnomAD) v2.1.1 [34]. ▪ The conventional pathogenicity prediction of c.5584G>A (p.Gly1862Ser) was done based the reported nomenclature G1862S. However, this variant was predicted to cause aberrant splicing by broking the WT donor splice site (HSF-Pro), likely leading to a premature termination: p.G1862fs*4. Therefore, the predicted pathogenicity may not reflect the true variant impact. Residual pLDDT is based on the full-length WT ABCA4 AF2 model. pLDDT≤50 for Arg-1300 (based on the domain-specific model) and Ser-2255, indicating a lack of confidence, as indicated with ǂ, should be interpreted with caution. RMSD. (Å): The root-mean-square deviation. 0.0 < TM-score < 1, where 0 is 100% alignment [24]. RMSD and TM-score are based on the structural comparison of the predicted variant models with the predicted WT ABCA4 model. We did not set a threshold for pathogenicity but showed the less well-aligning structures in this dataset in bold. Destabilizing variants were predicted using Gibbs free energy change calculation in the FoldX plugin for YASARA [46, 47]. Side chain steric clashes were reported here only when all possible rotamers led to clashing interactions. It was given as "mild" when all rotamers produced clashes, but at least one rotamer showed less severe clashing. ECD: Exocytoplasmic domain, NBD: Nucleotide-binding domain, TMD: Transmembrane domain. IH: intracellular α-helix. VUS: Variants of uncertain significance. Neu: Neutral, Del: Deleterious. PDB IDs of the cryo-EM structures of the human ABCA4 used in the analyses: 7lkp,7lkz,7e7o, 7m1q [30–32]. 20. Sun 2000, 21. Biswas 2003, 25. Garces 2020, 26. Curtis 2020, 27. Wiszniewski 2005, 28.Biswas et al 2010, 43. Scortecci 2021.

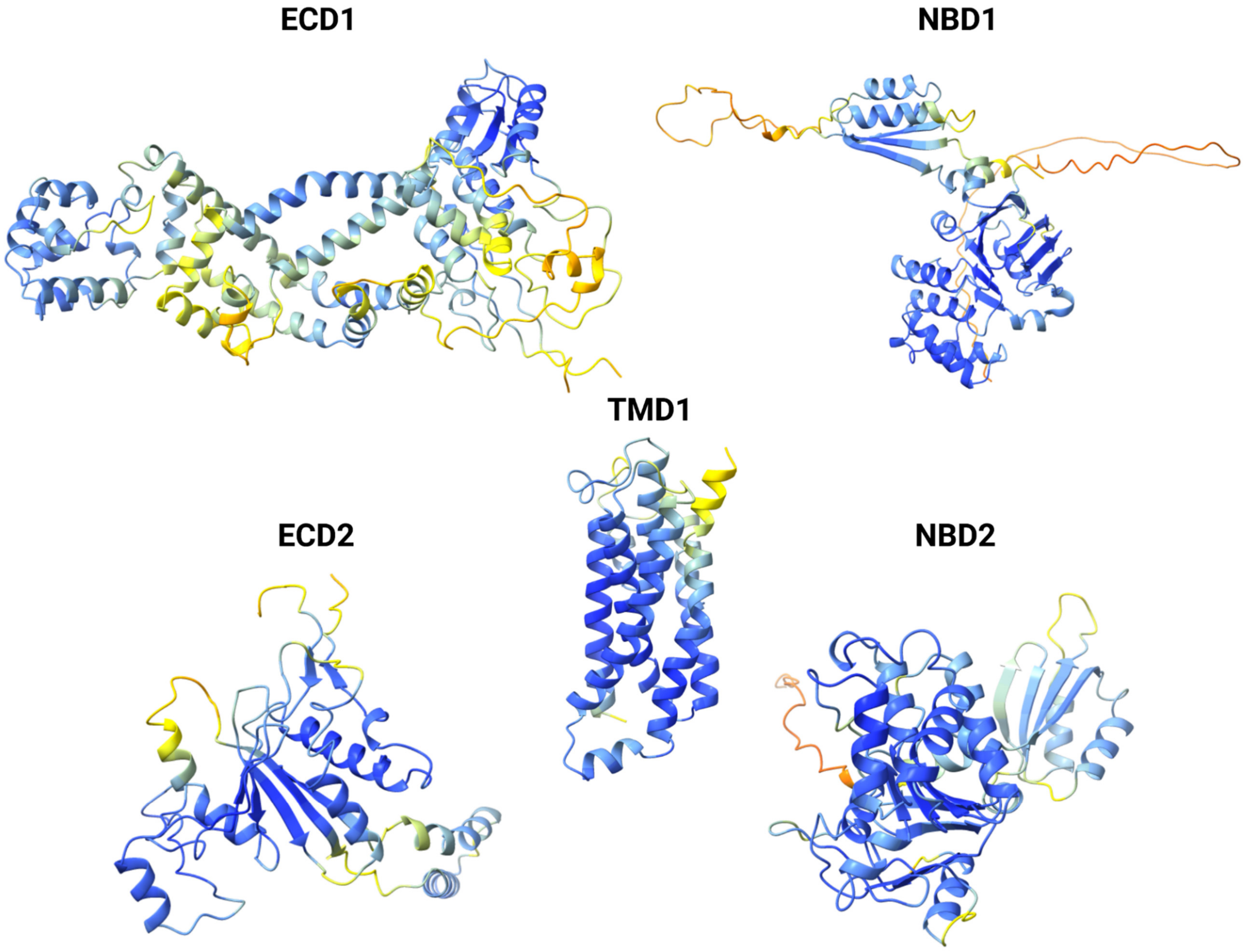

Figure S1. Domain-specific WT ABCA4 models generated in the AphaFold2 original Colab notebook from Deepmind [65]. The models were colored in the ChimeraX [67] using the “color bfactor palette alphafold” command to color the predicted structures by the pLDDT confidence measure.

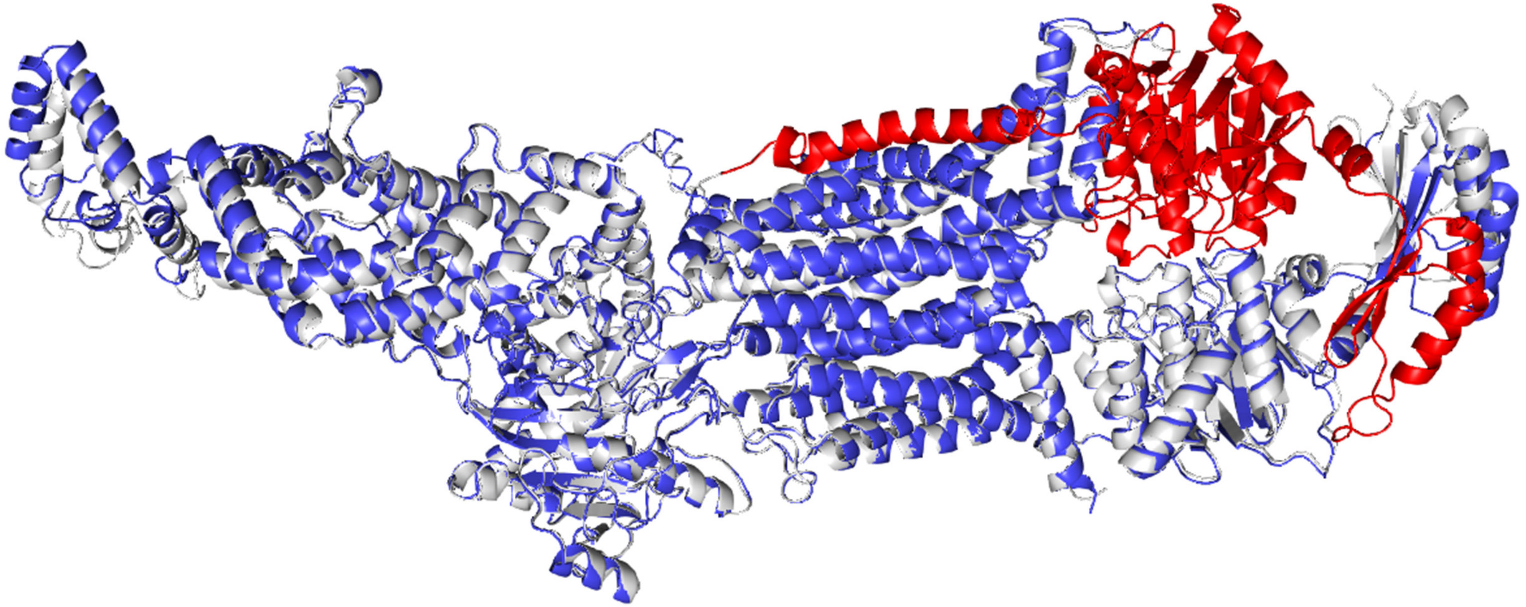

Figure S2. Predicted model of the c.5584G>A:p(Gly1862Ser) variant. The predicted effect of this variant is a truncated protein product (blue) superimposed onto the WT ABCA4 model (gray). The missing part of the protein is colored red in the WT model to illustrate the extent of the structural loss. The truncated protein lacks the last helix of the TMD2 and the entire NBD2 domain.
